# Supplementary material for: Phylogenomics of trans-Andean tetras of the genus Hyphessobrycon Durbin 1908 (Stethaprioninae: Characidae) and colonization patterns of Middle America
Source: PLoS One. 2023 Jan 20;18(1):e0279924. doi: 10.1371/journal.pone.0279924 (PMC9858358; doi:10.1371/journal.pone.0279924)
Supplement: S1 Fig — A) Inferred phylogeny based on the 75% complete data matrix B) inferred phylogeny based on the 90% complete data matrix, and C) inferred phylogeny based on the 75% complete data matrix. All nodes are supported with ultrafast bootstrap (UFBoot2) = 100 and SH-like approximate ratio test (SH-aLRT) = 100 unless noted. Nodes with gray circles UFBoot2 < 90 and SH-aLRT < 90. Species names with asterisk indicates samples from Melo et al. [9]. (PDF) [file pone.0279924.s001.pdf]

Supporting information - S1 Figure

Phylogenomics of *trans*-Andean tetras of the genus *Hyphessobrycon* Durbin 1908 (Stethaprioninae: Characidae) and colonization patterns of Middle America

Diego J. Elías[1,2*], Caleb D. McMahan[2], Fernando Alda[3,4], Carlos García-Alzate[5], Pamela B. Hart[1,6], Prosanta Chakrabarty[1]

[1]Museum of Natural Science, Department of Biological Sciences, Louisiana State University, Baton Rouge, Louisiana, United States of America

[2]Field Museum of Natural History, Chicago, Illinois, United States of America

[3]Department of Biology, Geology and Environmental Science, University of Tennessee at Chattanooga, Chattanooga, Tennessee, United States of America

[4] SimCenter: Center for Excellence in Applied Computational Science and Engineering, University of Tennessee at Chattanooga, Chattanooga, Tennessee, United States of America

[5]Grupo de Investigación Estudios en Sistemática y Conservación, Universidad del Atlántico-Corporación Universitaria Autónoma del Cauca, Colombia

[6] Department of Biological Sciences, The University of Alabama, Tuscaloosa, AL, United States of America

*Corresponding author:

E-mail: delias@fieldmuseum.org

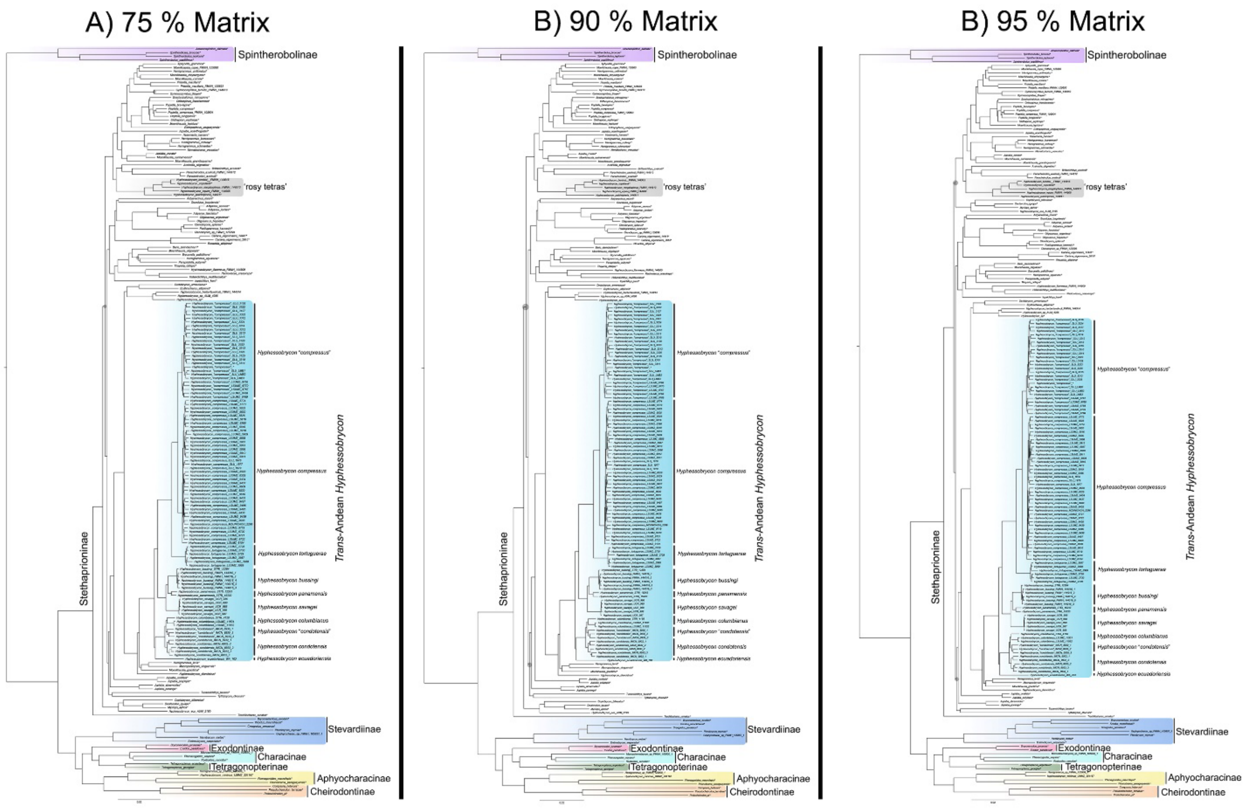

S1 Figure. Phylogenomic relationships of *trans*-Andean *Hyphessobrycon* based on concatenated analysis of ultraconserved elements. A) Inferred phylogeny based on the 75% complete data matrix B) inferred phylogeny based on the 90% complete data matrix, and C) inferred phylogeny based on the 75% complete data matrix. All nodes are supported with ultrafast bootstrap (UFBoot2) = 100 and SH-like approximate ratio test (SH-aLRT) = 100 unless noted. Nodes with gray circles UFBoot2 < 90 and SH-aLRT < 90. Species names with asterisk indicates samples from Melo et al. [9].

References

Melo BF, Sidlauskas BL, Near TJ, Roxo FF, Ghezelayagh A, Ochoa LE, et al. Accelerated Diversification Explains the Exceptional Species Richness of Tropical Characoid Fishes. Syst Biol. 2021;71: 78–92.
