# Supplementary material for: Phylogenomics of trans-Andean tetras of the genus Hyphessobrycon Durbin 1908 (Stethaprioninae: Characidae) and colonization patterns of Middle America
Source: PLoS One. 2023 Jan 20;18(1):e0279924. doi: 10.1371/journal.pone.0279924 (PMC9858358; doi:10.1371/journal.pone.0279924)
Supplement: S2 Fig — Gene concordance factors (gCF) and normalized quartet scores (NQS) for the main topology/quartet (A) and the two alternative quartets (B & C) for the inferred sister relationships of trans-Andean Hyphessobrycon. A) trans-Andean Hyphessobrycon sister to a clade comprise of M. gracilima, H. levis, and M. xinguensis (see Fig 2). B) trans-Andean Hyphessobrycon sister to H. diancistrus. C) trans-Andean Hyphessobrycon sister other characins of the sub-family Stethaprioninae. Number of trees decisive for the branch (gN) = 1646, effective number of genes for a branch of interest (EN) = 1220.72. gCF = gene concordance factor, gDF1 = gene discordance factor for NNI-1 branch, gDF2 = gene discordance factor for NNI-2 branch, gene discordance factor due to polyphyly (gDFP) = 65.37. NQS = normalized quartet score. (PDF) [file pone.0279924.s002.pdf]

Supporting information - S2 Figure

Phylogenomics of *trans*-Andean tetras of the genus *Hyphessobrycon* Durbin 1908 (Stethaprioninae: Characidae) and colonization patterns of Middle America

Diego J. Elías[1,2*], Caleb D. McMahan[2], Fernando Alda[3,4], Carlos García-Alzate[5], Pamela B. Hart[1,6], Prosanta Chakrabarty[1]

[1]Museum of Natural Science, Department of Biological Sciences, Louisiana State University, Baton Rouge, Louisiana, United States of America

[2]Field Museum of Natural History, Chicago, Illinois, United States of America

[3]Department of Biology, Geology and Environmental Science, University of Tennessee at Chattanooga, Chattanooga, Tennessee, United States of America

[4] SimCenter: Center for Excellence in Applied Computational Science and Engineering, University of Tennessee at Chattanooga, Chattanooga, Tennessee, United States of America

[5]Grupo de Investigación Estudios en Sistemática y Conservación, Universidad del Atlántico-Corporación Universitaria Autónoma del Cauca, Colombia

[6] Department of Biological Sciences, The University of Alabama, Tuscaloosa, AL, United States of America

*Corresponding author:

E-mail: delias@fieldmuseum.org

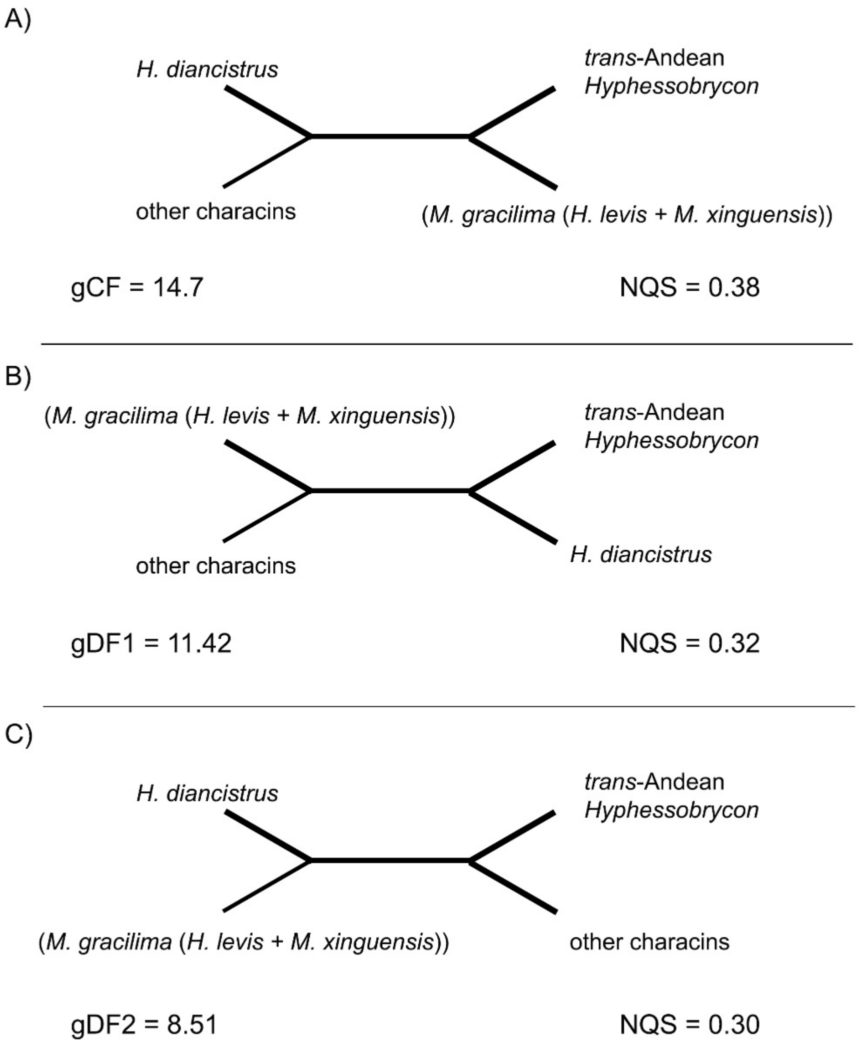

S2 Figure. Gene concordance factors (gCF) and normalized quartet scores (NQS) for the main topology/quartet (A) and the two alternatives quartets (B & C) for the inferred sister relationships of *trans*-Andean *Hyphessobrycon*. A) *trans*-Andean *Hyphessobrycon* sister to a clade comprise of *M. gracilima*, *H. levis*, and *M. xinguensis* (see Fig. 2) B) *trans*-Andean *Hyphessobrycon* sister to *H. diancistrus*, and C) *trans*-Andean *Hyphessobrycon* sister other characins of the subfamily Stethaprioninae. Number of trees decisive for the branch (gN) = 1646, effective number of genes for a branch of interest (EN) = 1220.72. gCF = gene concordance factor, gDF1 = gene discordance factor for NNI-1 branch, gDF2 = gene discordance factor for NNI-2 branch, gene discordance factor due to polyphyly (gDFP) = 65.37. NQS = normalized quartet score.
