# Supplementary material for: Phylogenomics of trans-Andean tetras of the genus Hyphessobrycon Durbin 1908 (Stethaprioninae: Characidae) and colonization patterns of Middle America
Source: PLoS One. 2023 Jan 20;18(1):e0279924. doi: 10.1371/journal.pone.0279924 (PMC9858358; doi:10.1371/journal.pone.0279924)
Supplement: S2 Table — LnL = log-likelihood score for the model. k = number of parameters, d = dispersal rate, e = extinction rate or range loss rate, j = jum-dispersal rate, AICc = Akaike information criterion corrected. AICc wt = Akaike information criterion corrected weighted. The best biogeographic model was selected based on the highest AICc wt, in bold. (PDF) [file pone.0279924.s004.pdf]

Supporting information - S2 Table

Phylogenomics of *trans*-Andean tetras of the genus *Hyphessobrycon* Durbin 1908 (Stethaprioninae: Characidae) and colonization patterns of Middle America

Diego J. Elías[1,2*], Caleb D. McMahan[2], Fernando Alda[3,4], Carlos García-Alzate[5], Pamela B. Hart[1,6], Prosanta Chakrabarty[1]

[1]Museum of Natural Science, Department of Biological Sciences, Louisiana State University, Baton Rouge, Louisiana, United States of America

[2]Field Museum of Natural History, Chicago, Illinois, United States of America

[3]Department of Biology, Geology and Environmental Science, University of Tennessee at Chattanooga, Chattanooga, Tennessee, United States of America

[4] SimCenter: Center for Excellence in Applied Computational Science and Engineering, University of Tennessee at Chattanooga, Chattanooga, Tennessee, United States of America

[5]Grupo de Investigación Estudios en Sistemática y Conservación, Universidad del Atlántico-Corporación Universitaria Autónoma del Cauca, Colombia

[6] Department of Biological Sciences, The University of Alabama, Tuscaloosa, AL, United States of America

*Corresponding author:

E-mail: delias@fieldmuseum.org

S2 Table Parameters estimates of six different biogeographic models. LnL = log-likelihood score for the model. *k* = number of parameters, *d* = dispersal rate, *e* = extinction rate or range loss rate, *j* = jum-dispersal rate, AICc = Akaike information criterion corrected. AICc wt = Akaike information criterion corrected weighted. The best biogeographic model was selected based on the highest AICc wt, in bold.

Model	LnL	*k*	*d*	*e*	*j*	AICc	AICc wt
**DEC-like**	-25.52	2	0.57	0.43	0	**56.24**	**0.36**
DEC-like + J	-24.58	3	0.091	1.00 e^{-12}	0.031	57.83	0.16
DIVA-like	-25.99	2	0.17	1.00 e^{-12}	0	57.18	0.22
DIVA-like + J	-24.19	3	0.10	1.00 e^{-12}	0.028	57.05	0.24
BAYAREA-like	-37.36	2	0.15	0.21	0	79.93	2.60 e^{-06}
BAYAREA-like + J	-27.11	3	0.079	1.00 e^{-07}	0.054	62.89	0.013
